# Indigenous Eye Health in the Americas: The Burden of Vision Impairment and Ocular Diseases

**DOI:** 10.3390/ijerph20053820

**Published:** 2023-02-21

**Authors:** João Marcello Furtado, Arthur Gustavo Fernandes, Juan Carlos Silva, Sandra Del Pino, Carolina Hommes

**Affiliations:** 1 Pan American Health Organization, Washington, DC 20037, USA; 2 Division of Ophthalmology, Ribeirão Preto Medical School, University of São Paulo, Ribeirao Preto 14015-010, Brazil; 3 Department of Ophthalmology and Visual Sciences, Federal University of São Paulo, Sao Paulo 04023-062, Brazil; 4 Department of Anthropology and Archaeology, University of Calgary, Calgary, AB T2N 4N1, Canada; 5 Independent Researcher, Bogota 110111, Colombia

**Keywords:** health services accessibility, Indigenous health service, Indigenous Peoples, ophthalmology, public health

## Abstract

Review of the burden of vision impairment and blindness and ocular disease occurrence in Indigenous Peoples of the Americas. We systematically reviewed findings of the frequency of vision impairment and blindness and/or frequency of ocular findings in Indigenous groups. The database search yielded 2829 citations, of which 2747 were excluded. We screened the full texts of 82 records for relevance and excluded 16. The remaining 66 articles were examined thoroughly, and 25 presented sufficient data to be included. Another 7 articles derived from references were included, summing a total of 32 studies selected. When considering adults over 40 years old, the highest frequencies of vision impairment and blindness in Indigenous Peoples varied from 11.1% in high-income North America to 28.5% in tropical Latin America, whose rates are considerably higher than those in the general population. Most of the ocular diseases reported were preventable and/or treatable, so blindness prevention programs should focus on accessibility to eye examinations, cataract surgeries, control of infectious diseases, and spectacles distribution. Finally, we recommend actions in six areas of attention towards improving the eye health in Indigenous Peoples: access and integration of eye services with primary care; telemedicine; customized propaedeutics; education on eye health; and quality of data.

## 1. Introduction

Vision impairment and blindness are estimated to affect more than 339 million people worldwide, with 43.3 million people blind and 295.3 million people having moderate to severe visual impairment (MSVI), representing a prevalence of 5.25 cases of blindness per 1000 persons (95% CI: 4.58–5.87) and 35.8 cases of MSVI per 1000 persons (95% CI: 32.4–39.2) [1]. Cataract, glaucoma, under-corrected refractive errors, age-related macular degeneration, and diabetic retinopathy are the main causes of blindness, while the main causes of MSVI are uncorrected refractive errors, cataract, age-related macular degeneration, glaucoma, and diabetic retinopathy [2]. In the Americas, the estimates vary substantially across the different Global Burden of Disease (GBD) regions, with blindness estimates ranging from 1.93 cases per 1000 people in southern Latin America (i.e., Argentina, Chile, and Uruguay) to 7.40 cases per 1000 in tropical Latin America (i.e., Brazil and Paraguay) [1].

Most global estimates, however, do not include data from Indigenous Peoples and other ethnic groups, even though those groups are expected to present higher frequencies of ocular diseases and vision loss [3,4,5]. As a result, the burden of vision impairment and blindness may be underestimated, and the public health policies derived from it may insufficiently attend the demand in those minority groups. Including those groups in population-based sample sizes is often challenging due to the low number of individuals in comparison with the overall population and/or due to the low response from those specific groups even when they are included in the sampling [6,7]. Developing and implementing services designed to prioritize reaching groups in situations of vulnerability, as with Indigenous Peoples, with quality and affordable eye services was recently listed as one of the main challenges in global eye health [8].

Indigenous individuals can, on certain occasions, be considered one of the most disadvantaged and marginalized populations worldwide [9]. A recent systematic review of vision loss among Indigenous populations has shown a lack of data on the burden of vision loss in most countries and has pointed out the importance of improving the quality and number of research about eye health and eye care in Indigenous communities [10]. Different Indigenous groups from different nations have unique characteristics in language, culture, environmental risk factors, and political autonomy, yet, as a result of the colonization process, many face similar health disparities and social disadvantages [11]. Indigenous groups currently account for around 17% of those living in extreme poverty in Latin America, even though they represent less than 8% of the population [12].

It is estimated that in 2010 there were at least 44.8 million Indigenous persons in Latin America, representing 826 Indigenous Peoples mainly concentrated in Mexico (seventeen million people) and Peru (seven million), followed by Guatemala and Bolivia (six million in each) [13]. While they are the majority of the population in Bolivia (62%) and Guatemala (60%), they represent less than 2% in Brazil, Colombia, Venezuela, and the Caribbean [14,15]. In the United States (USA), 6.6 million Alaskan Natives and Native American Indians (2% of the general population) live in 567 tribes, and 326 Indian reserves are officially recognized by the federal government [16]. In Canada, 5% of the total population is identified as Indigenous, summing 1.8 million individuals from First Nations, Métis, and Inuit groups [17].

The purpose of the current study is to conduct a review of the burden of vision impairment and blindness and ocular disease occurrence in the Indigenous Peoples of the Americas while comparing it to the estimates based on non-indigenous populations and identifying gaps in the literature.

## 2. Materials and Methods

We systematically reviewed findings on frequencies of vision impairment and blindness or frequencies of ocular findings such as cataract, under-corrected refractive errors, glaucoma, age-related macular degeneration, diabetic retinopathy, pterygium, trachoma, and onchocerciasis in Indigenous populations in Americas. We searched for any study evaluating eye health not limiting the sources to population-based data. The search combined terms related to three concept areas: population (Indigenous), outcome (vision impairment/blindness and ocular findings), and study site (the Americas). Term selection was based on previous systematic reviews and combined key terms adapted for each database and medical subject headings (MeSH) as applicable. We searched for studies in any language, indexed from 1 January 2000 to 1 November 2022.

We screened the selected papers in terms of (1) reporting frequencies of vision impairment or blindness or frequencies of ocular diseases; (2) reporting results for an indigenous population; and (3) reporting data from populations resident in any region of the Americas. We excluded articles that did not include an Indigenous group, were iterations, were program evaluations or experimental studies, not primary studies, were from the gray literature, or used identical data sources as prior studies. Because many studies on Indigenous Peoples have not reported response rates, we did not impose any minimum response rate limit. Self-reported outcome data were not included.

The following information was extracted from each selected study: author, year of publication, country, Indigenous group, study design, sample size, individuals age, main outcome, method for visual acuity and definitions for vision impairment and blindness, frequency of vision impairment and blindness, and/or frequency of ocular diseases. The results were presented separately according to the GBD regions classification (Table 1).

We presented the results as descriptive tables for frequencies of vision impairment and blindness and for frequencies of ocular diseases in the population. As most of the studies adopted different criteria for definitions of vision impairment and blindness and varied the measurement method (i.e., uncorrected, presenting vision, and best-corrected vision acuity), we could not standardize estimates and summarize the findings per region and therefore presented descriptive data along with the specificities of each estimate.

## 3. Results

**Figure 1:**
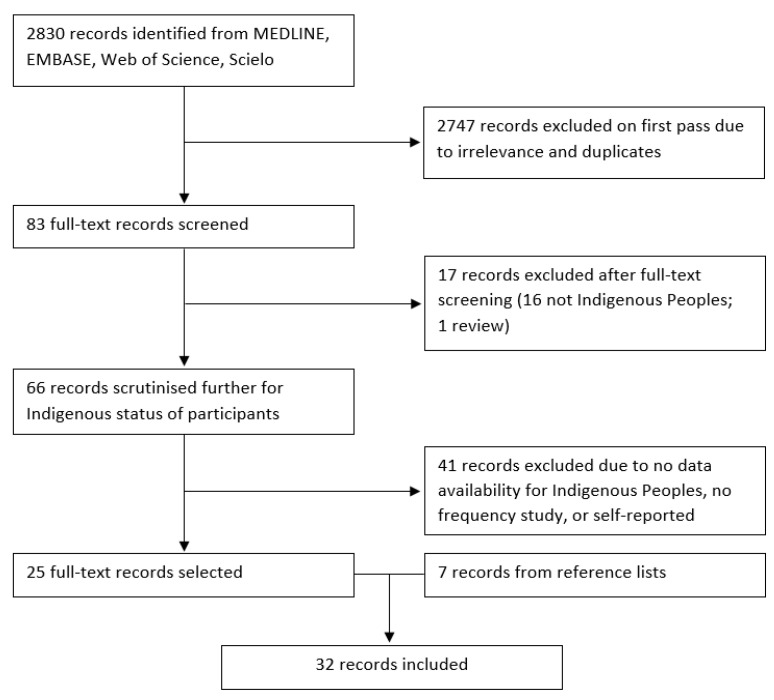
Systematic search and selection of eligible studies.

The database search yielded 2829 citations, of which 2747 were excluded. We screened the full texts of 82 records for relevance and excluded 16. The remaining 66 articles were examined thoroughly, and 25 presented sufficiently data to be included in the current review. Another 7 articles derived from references were included, summing a total of 32 studies selected. Figure 1 shows the flowchart of records selection.

Out of the 32 selected studies, 14 (43.75%) were conducted in tropical Latin America (13 in Brazil and 1 in Paraguay), 12 (37.50%) in high-income North America (8 in the USA and 4 in Canada), 4 (12.50) in central Latin America (2 in Colombia, 1 in Mexico, and 1 in Venezuela), 1 (3.12%) in Andean Latin America (Ecuador), and 1 (3.12%) in the Caribbean (Haiti). No studies from southern Latin America were included.

A total of 11 studies (34.37%) reported frequencies of vision impairment and blindness, with most of them from high-income North America. No studies from Andean Latin America, the Caribbean, or southern Latin America presented data on vision impairment and blindness. A great variability of vision acuity measurement methods, as well as vision impairment and blindness definitions, was observed. Table 2 shows the frequencies of vision impairment and blindness according to the GBD region along with study population Indigenous group and age, and categories’ definitions.

Despite the differences in the vision impairment and blindness definitions, it is a clear significant difference in the frequencies between high-income North America and tropical Latin American countries. When considering adults over 40 years old and the BCVA method, the highest frequencies of vision impairment and blindness in high-income North America sum 11.1% [27] while in the tropical Latin America it can reach 28.5% [19].

A total of 26 studies (81.25%) reported frequencies of ocular diseases, with most of them from tropical Latin America and high-income North America. Trachoma was the main condition evaluated, discussed in nine studies (34.61%), with six in tropical Latin America and three in Central America. Cataract was evaluated in seven studies (26.92%), three in high-income North America, three in tropical Latin America, and one in the Caribbean. Interestingly, the six studies evaluating diabetic retinopathy (23.07%) were from high-income North America. Pterygium was evaluated in five studies (19.23%), with four from tropical Latin America and one from the Caribbean. Table 3 shows the frequencies of ocular diseases according to the GBD region along with study population Indigenous group and ages.

## 4. Discussion

This study presents an overall panorama of the ocular health in Indigenous Peoples in the America. The main limitation, however, is the shortage of data. The low number of records retrieved from our literature review reflects the scarcity of studies focused on eye health in Indigenous populations in the Americas. Out of the 33 countries in the Americas, only 7 (21%) had data on vision impairment/blindness and/or ocular disease in Indigenous groups. The lack of studies is particularly more evident in Andean Latin America, where a high percentage of the population self-identify as Indigenous and yet is underrepresented [14]. No studies were found for southern Latin America, which is the sub-region with the lowest frequencies of Indigenous Peoples in the general population. The most recent worldwide estimates of vision impairment and blindness, however, have included data from most countries in the Americas, indicating availability of population-based surveys and therefore reinforcing the misrepresentation of Indigenous Peoples in these calculations. While part of these studies might have included Indigenous groups in their samples, most of them have used the RAAB (Rapid Assessment of Avoidable Blindness) methodology, which is a format that does not disaggregate information on ethnicity further limiting the analysis of burden of disease in Indigenous populations specifically and the comparisons between Indigenous and non-indigenous groups [50].

Most studies on frequency of vision impairment and blindness were conducted in high-income North America. According to the GBD, the prevalence of moderate to severe vision impairment (MSVI: VA < 20/63 to VA ≥ 20/400) and blindness (VA < 20/400) in the general population aged 50 years and older in the region was 3.28% and 0.40%, respectively [1]. Despite the different criteria for classification, the frequency of vision impairment and blindness in the Indigenous populations evaluated were higher than those presented by the GBD, with values in older adults ranging from 3.10% [28] to 12.80% [25] for vision impairment and 0.30% [28] to 1.90% [27] for blindness.

Tropical Latin America is one of the sub-regions with the highest estimated rates of MSVI (10.60%) and blindness (2.71%) in older adults in the Americas [1]. A recent study performed with residents from the Xingu Indigenous Park in Brazil following the same GDB criteria of classification has shown frequencies of MSVI and blindness substantially higher than those calculated for the general population, reaching 22.58% and 5.92%, respectively, in adults 45 years and older [19].

The only study from central Latin America evaluated individuals 20 years and older in Mexico and found a prevalence of presenting vision acuity <20/60 in 10% of the population [18]. The estimates for MSVI and blindness considering best-corrected vision acuity in adults 50 years and older in the region were 10.70% and 1.83% [1], but due to the different criteria of measurement and definitions, we are not able to make direct comparisons.

The general estimates of vision impairment and blindness for Andean Latin America (MSVI: 13.00%; blindness: 2.20%), the Caribbean (MSVI: 8.22%; blindness: 1.74%), and southern Latin American (MSVI: 6.59%; blindness: 0.66%) could not be compared to Indigenous Peoples due to the lack of studies on these groups in those specific countries [1].

In 2020, cataract and under-corrected refractive error composed 50% of all global blindness and 75% of all global MSVI [2]. Other causes included glaucoma, age-related macular degeneration, and diabetic retinopathy, being the five conditions mostly studied in the general population. Diabetic retinopathy was the smallest contributor to blindness in 2020 among those, however, it was the only cause of blindness that showed a global increase in prevalence from 1990 to 2020, particularly in the high-income North America sub-region [2]. While the data retrieved from studies using Indigenous populations cover extensive age ranges and do not necessarily represent the disease frequency or the cause of MSVI and blindness, a differential pattern of disease focus is observed among the sub-regions. While 66.7% of the studies from high-income North America have presented data on diabetic retinopathy, none of the studies from the other region have evaluated this condition.

The cataract rates in older adults, regardless of vision acuity status, have varied from 12.2% in Northwestern and Alaskan Natives in the USA [28] to 54.5% in groups from the Xingu Indigenous Park in Brazil [19]. These values are sensitive to the population’s access to cataract surgeries, which may explain the high frequency of disease in Indigenous populations with limited access to specialized eye health services. Few studies evaluated refractive errors, with rates reaching up to 62% in Brazilian communities [43]. The effective cataract surgical coverage (eCSC) and the effective refractive error coverage (eREC) are indicators requested by the WHO in order to meet the 2030 Sustainable Development Goals [51]. eCSC refers to the proportion of people who have received cataract surgery and have a resultant good quality outcome relative to the number of people in need of cataract surgery [52]. Similarly, eREC refers to the proportion of people who have received refractive correction and have a resultant good quality outcome relative to the number of people in need of refractive correction [53]. These indicators are ideal to not only track changes in the uptake and quality of eye care services, but also to contribute to monitoring progress towards universal health care in general [54]; however, none of the studies using Indigenous populations in the Americas have reported eCSC or eREC. A previous analysis of Indigenous versus non-indigenous groups in Australia has shown that eCSC was significantly better in non-indigenous Australians than in Indigenous Australians (88.5% vs. 51.6%) [55].

Pterygium is a condition commonly evaluated in the studies as its occurrence is associated with geographic locations characterized by low latitude and high ultraviolet exposure. In that sense, studies from the Caribbean, central and tropical Latin America have reported frequencies from 12.8% [40] to 27.1% [43]. The population profile is a determinant for pterygium development, so people who have an outdoor lifestyle tend to be more likely to develop the disease due to the direct UV exposure. The disease is also highly prevalent in non-indigenous populations in equatorial areas with prevalence reaching up to 58.8% [56].

Ocular infectious diseases are highly associated with living style, access to clean water, and basic sanitation, and therefore can be highly prevalent in Indigenous communities [57]. Trachoma and onchocerciasis were evaluated in 73% of the studies from central and tropical Latin America reflecting the concern about such conditions in these regions. Onchocerciasis was identified in two studies in Brazil, affecting up to 68.6% of a Yanomami community [35]. Trachoma was identified in both central and tropical Latin America with frequencies ranging from 6.9% [31] to 41.8% [34]. Moreover, one study in Brazil evaluated parasitic keratitis in Arawak, Tukano, and Maku peoples finding a frequency of 17.2% [43].

Historically, onchocerciasis was formerly prevalent in 13 foci in Brazil, Colombia, Ecuador, Guatemala, Mexico, and Venezuela [58]. In response, the Pan American Health Organization (PAHO) established the Onchocerciasis Elimination Program for the Americas (OEPA) in 1992 with the main purpose to guide countries to achieve the goal of eliminating onchocerciasis in Latin America [59]. In general, the strategy included six-monthly mass administration of ivermectin (Mectizan^®^, Merck & Co. Inc., Rahway/NJ, USA) with coverage equal to or higher than 85% of the eligible population [59]. The onchocerciasis elimination program in Latin American countries has been ongoing since 1996 [60]. To date, onchocerciasis transmission has been eliminated from 11 of the 13 previously endemic disease foci in Latin America, and four out of six endemic countries have been verified as eliminated by PAHO (Colombia, Ecuador, Guatemala, and Mexico) [61].

Trachoma is the world’s leading infectious cause of blindness and is endemic in several parts of the world [62]. Mexico was the first country in the Americas to eliminate trachoma as a public health problem, as validated by PAHO in 2017, but this is still a concern in four countries in Andean, central, and tropical Latin America: Brazil, Colombia, Guatemala, and Peru [63,64]. PAHO/WHO support countries to implement the SAFE strategy (i.e., surgery, antibiotics, facial cleanliness, and environmental improvement), a program that consists of surgery to treat advanced trachoma (trichiasis), antibiotics (azithromycin) to clear the agent of infection, facial hygiene, and environmental improvements to reduce transmission from one person to another [63]. While the strategy adherence might be more challenging in Indigenous communities, the example from Mexico reinforces the importance of partnership with local leader authorities who will enhance the population’s trust in the program and improve the outcomes [64].

Other conditions observed in the reviewed studies include glaucoma and under-corrected refractive errors. Glaucoma was present in a relatively small proportion of the populations of Brazil and the USA but at a high frequency of 19.1% in Haiti [30]. The high frequency of glaucoma in Haiti could be influenced by the nonvariation in race and the higher environment temperature [30,65]. In general, the high rates of cataract and under-corrected refractive errors reflect the poor access of the Indigenous populations to specialized care. The access is likely associated with education and economic status, which are factors that could not be evaluated in the current revision due to the lack of information in the selected studies [66,67].

There are significant disparities in the number and distribution of ophthalmologists in American countries as they tend to be concentrated in more developed cities, leaving remote areas, where most Indigenous Peoples are concentrated, with a low density of ophthalmologists [66]. Due to a lack of access to and utilization of eye care services, Indigenous Peoples in the Amazon may combine several social determinants of blindness and visual impairment, such as ethnicity, place of residence (rural remote areas), socioeconomic status (poverty), and education (low levels of schooling). In Guatemala, with a high percentage of Indigenous population and high prevalence of blindness [67], the determinant “place of living” might not be as important as in the Amazon, but others are present among Indigenous groups. More recently, social, political, and economic crises have motivated intense migratory movements and refugee requests in Latin American countries, with an increasing number of Indigenous individuals living in public or self-managed shelters or even on the street in extreme poverty. These conditions represent an extra challenge to address, not only visual, but the general health care needs of such groups [68,69,70].

Improving Indigenous eye health in the Americas is particularly challenging and mainly due to limited access and inequalities in care. More than achieving universal health coverage in a country, equity should be prioritized, otherwise, socially advantaged groups will be more likely to use the new or improved services [71,72]. Specific actions include the following: (1) access: increasing the number of clinic sites, rural locations, and eye care sessions, not only with ophthalmologists, but also with other eye health practitioners as optometrists, ophthalmic technologists, and/or trained nurses should improve the number of patient seen, dispensing spectacles, and surgery referrals [72,73]; (2) integration with family medicine/primary care: several communities have general health programs with systemic condition screening and could include ocular health screening tools into their practice to detect and timely refer cases of vision impairment and blindness for specialized care [19,72,74]; (3) telemedicine: several telemedicine protocols in ophthalmology focused on diabetes retinopathy, glaucoma, and cataract have been shown to be effective in populations living in remote areas and should be used as models towards Indigenous population groups [75,76,77]; (4) customized propaedeutics: specific techniques should be indicated to populations living in remote areas, for example, manual small incision cataract surgery (MSICS) techniques in resource-constrained health care settings such as Indigenous communities [78]; (5) education on eye health: by promoting basic knowledge on eye health, the population can better understand the importance of seeking timely treatment, improving visual outcomes [79,80]; (6) quality data: more studies focused on Indigenous population’s eye health should be performed with appropriate methodology and collection of key indicators such as eCSC and eREC, and studies performed in the general population should collect data on the participants’ ethnicity/race [52,53].

## 5. Conclusions

Despite the shortage of data, our findings show a higher frequency of vision impairment and blindness in the Indigenous population when compared to worldwide estimates for all sub-regions in the Americas. Most of the ocular diseases reported are preventable and/or treatable, so blindness prevention programs should focus on accessibility to eye examinations, cataract surgeries, control of infectious diseases, and spectacles distribution. Finally, more epidemiological studies with Indigenous populations using higher methodologic quality and consistent indicators are recommended in order to understand the burden of diseases and optimize developed programs focused on these groups.

## Figures and Tables

**Table 1:** Countries in the Americas by region.

Region	Countries
Andean Latin America	Bolivia, Ecuador, Peru
The Caribbean	Antigua and Barbuda, The Bahamas, Barbados, Belize, Bermuda, Cuba, Dominica, Dominican Republic, Grenada, Guyana, Haiti, Jamaica, Puerto Rico, Saint Lucia, Saint Vincent and the Grenadines, Suriname, Trinidad and Tobago
Central Latin America	Colombia, Costa Rica, El Salvador, Guatemala, Honduras, Mexico, Nicaragua, Panama, Venezuela
High-Income North America	Canada, Greenland, United States of America
Southern Latin America	Argentina, Chile, Uruguay
Tropical Latin America	Brazil, Paraguay

**Table 2:** Frequency of vision impairment and blindness.

Author, Year	Country	Indigenous Group	Study Design	Sample Size	Age	V.A. method	V.I. Definition	Prevalence V.I.(95% CI)	Blindness Definition	Prevalence Blindness (95%CI)
**Andean Latin America**										
No data available										
**Caribbean**										
No data available										
**Central Latin America**										
Corona, 2015 [18]	Mexico	Non-Specified	Survey	512	20+	PVA	VA < 20/60 toVA ≥ 20/200	10.00 (6.90–14.40)	VA ≤ 20/200	0.00
**Tropical Latin America**										
Fernandes, 2021 [19]	Brazil	Kalapalo, Kamaiura, Ikpeng, Yawalapiti	Survey	2099	0–17	BCVA	VA < 20/32 toVA ≥ 20/400	0.24	VA < 20/400	0.03
					18–44			1.84		0.25
					45+			22.58		5.92
Carter, 2013 [20]	Paraguay	Macca	Survey	117	0–17	PVA	VA < 20/40	5.98		
Salum, 2012 [21]	Brazil	Kadiweus	Survey	171	all ages	PVA	VA < 20/25	25.15		
Rehder, 2001 [22]	Brazil	Bororo, Karaja, Xavantes	Survey	900	1–94	BCVA	VA < 20/40 toVA ≥ 20/200	2.00	VA ≤ 20/200	2.67
**High-Income North America**										
Woodward, 2021 [23]	USA	American Indian, Native Alaskan, Native Hawaiian, and Pacific Islander	Survey	177,100	all ages	BCVA	≤20/32	1.48 (1.42–1.53)		
Aljied, 2018 [24]	Canada	Non-Specified	Survey	357	45–84	BCVA	VA < 20/40	6.50		
McClure, 2009 [25]	USA	American Indian, Alaska Natives	Survey	414	40+	PVA	VA < 20/40 toVA > 20/200	12.80 (9.60–16.00)	VA ≤ 20/200	0.50 (0.10–1.54)
Harvey, 2006 [26]	USA	Tohono O’odham	Survey	1327	5–16	BCVA	VA < 20/40	34.90		
Lee, 2005 [27]	USA	Oklahoma Native	Survey	1019	48–59	BCVA	VA < 20/40 toVA ≥ 20/200	1.20	VA < 20/200	0.50
					60–69			1.90		0.50
					70–82			6.10		0.90
Mansberger, 2005 [28]	USA	Northwest and Alaska Native	Survey	288	40+	BCVA	VA < 20/32 toVA ≥ 20/200	3.10 (1.00–5.00)	VA < 20/200	0.30 (0.00–1.00)
**Southern Latin America**										
No data available										

Legend: VA—visual acuity; PVA—presenting visual acuity; BCVA—best-corrected visual acuity; V.I.—vision impairment; USA—United States of America; CI—confidence interval.

**Table 3:** Frequency of ocular diseases.

Author	Country	Indigenous Group	Study Design	Sample Size	Age	Disease	Frequency in the Population (95% CI)
**Andean Latin America**							
Del Brutto, 2021 [29]	Ecuador	Atahualpa	Survey	241	60+	Hypertensive Retinopathy	17.80
**Caribbean**							
Duong, 2012 [30]	Haiti	Lascahobas	Survey	3702	0–92	Under-corrected Refractive Error	53.27
						Cataract	26.50
						Glaucoma	19.07
						Pterygium	14.56
						Age-Related Macular Degeneration	1.32
**Central Latin America**							
Lopez, 2022 [31]	Venezuela	Yanomami	Survey	1182	1–9	Trachoma	6.92
Miller, 2020 [32]	Colombia	Cubea, Desana, Tucana, Guanana, Siriana	Survey	4529	1–9	Trachoma	23.90
Miller, 2010 [33]	Colombia	Maku	Survey	114	1+	Trachoma	18.42
**Tropical Latin America**							
					0–17	Cataract	2.86
					18–44		2.67
					45+		54.46
Fernandes, 2021 [19]	Brazil	Kalapalo, Kamaiura, Ikpeng, Yawalapiti	Survey	2099	0–17	Under-corrected Refractive Error	37.14
					18–44		50.00
					45+		21.28
					0–17	Pterygium	0.00
					18–44		13.00
					45+		26.30
Freitas, 2016 [34]	Brazil	General	Pooled data	9582	all ages	Trachoma	41.80
Herzog-Neto, 2014 [35]	Brazil	Yanomami	Survey	86	9–74	Onchocerciasis	68.60
Salum, 2012 [21]	Brazil	Kadiweus	Survey	177	all	Pterygium	14.69
Neto, 2009 [36]	Brazil	Yanomami	Survey	83	0–20	Onchocerciasis	12.10
					21–40		14.50
					41–60		8.40
Cruz, 2008 [37]	Brazil	Non-Specified	Survey	311	1–10	Trachoma	9.00
Piccinin, 2007 [38]	Brazil	Terena	Survey	226	10–45	Dichromatopsia	0.00
Paula, 2006 [39]	Brazil	Arawak, Tukano, Maku, Yanomami	Survey	624	18+	Cataract	18.27
						Pterygium	18.43
Reis, 2002 [40]	Brazil	Tukano, Maku	Survey	179	all ages	Trachoma	55.00
						Pterygium	12.80
Paula, 2002 [41]	Brazil	Yanomami	Survey	613	all ages	Trachoma	30.34
Alves, 2002 [42]	Brazil	Hupde, Tukano, Daw	Survey	333	all ages	Trachoma	44.44
Garrido, 2000 [43]	Brazil	Arawak, Tukano, Maku	Survey	395	all ages	Parasitic Keratitis	17.22
						Under-corrected Refractive Error	62.03
						Trachoma	32.66
						Pterygium	27.09
						Cataract	20.25
						Glaucoma	5.57
**High-Income North America**							
Fonda, 2022 [44]	USA	American Indians, Alaska Natives	Survey	53,900	20+	Diabetes Retinopathy	28.60 (28.20–29.00)
						Diabetic Macular Edema	3.00 (2.80–3.10)
Woodward, 2021 [23]	USA	American Indian, Native Alaskan, Native Hawaiian, and Pacific Islander	Survey	177,100	all ages	Under-corrected Refractive Error	17.21 (17.03–17.39)
						Cataract	30.06 (29.83–30.28)
						Age-Related Macular Degeneration	7.13 (6.99–7.26)
						Glaucoma	11.86 (11.70–12.02)
Maple-Brown, 2012 [45]	Canada	Oji-Cree	Survey	124	10+	Diabetes Retinopathy	25.00
Rudnisky, 2012 [46]	Canada	Non-Specified	Survey	980	14–92	Non-Proliferative Diabetes Retinopathy	18.26
						Proliferative Diabetes Retinopathy	2.45
Butt, 2011 [47]	USA	Oklahoma Native	Survey	986	48–59	Age-Related Macular Degeneration	30.60
					60–69		32.60
					70–82		46.10
McClure, 2011 [48]	USA	American Indian, Native Alaskan	Survey	102	18+	Under-corrected Refractive Error	25.49
Lee, 2005 [27]	USA	Oklahoma Native	Survey	1019	48–82	Cataract	39.60
						Age-Related Macular Degeneration	33.60
						Diabetes Retinopathy	20.10
						Glaucoma	5.60
						Pinguecula	42.40
						Dermatochalasis	30.10
Mansberger, 2005 [28]	USA	Northwest and Alaskan Native	Survey	288	40+	Under-corrected Refractive Error	18.10 (13.60–22.60)
						Age-Related Macular Degeneration	18.30 (12.50–24.10)
						Cataract	12.20 (8.30–16.10)
						Glaucoma	6.20 (2.60–7.80)
						Non-Proliferative Diabetes Retinopathy	4.20 (1.80–6.60)
						Proliferative Diabetes Retinopathy	1.80 (0.20–3.40)
Maberley, 2002 [49]	Canada	Cree	Survey	100	24–82	Non-Proliferative Diabetes Retinopathy	24.00
						Diabetic Macular Edema	5.00
						Proliferative Diabetes Retinopathy	2.00
**Southern Latin America**							
No data available							

Legend: USA—United States of America; CI—confidence interval.

## Data Availability

No new data were created or analyzed in this study. Data sharing is not applicable to this article.
